# Does higher education hone cognitive functioning and learning efficacy? Findings from a large and diverse sample

**DOI:** 10.1371/journal.pone.0182276

**Published:** 2017-08-23

**Authors:** Belén Guerra-Carrillo, Kiefer Katovich, Silvia A. Bunge

**Affiliations:** 1 Department of Psychology, University of California at Berkeley, Berkeley, CA, United States of America; 2 General Assembly Space, Inc., San Francisco, CA, United States of America; 3 Helen Wills Neuroscience Institute, University of California at Berkeley, Berkeley, CA, United States of America; University of Westminster, UNITED KINGDOM

## Abstract

Attending school is a multifaceted experience. Students are not only exposed to new knowledge but are also immersed in a structured environment in which they need to respond flexibly in accordance with changing task goals, keep relevant information in mind, and constantly tackle novel problems. To quantify the cumulative effect of this experience, we examined retrospectively and prospectively, the relationships between educational attainment and both cognitive performance and learning. We analyzed data from 196,388 subscribers to an online cognitive training program. These subscribers, ages 15–60, had completed eight behavioral assessments of executive functioning and reasoning at least once. Controlling for multiple demographic and engagement variables, we found that higher levels of education predicted better performance across the full age range, and modulated performance in some cognitive domains more than others (e.g., reasoning vs. processing speed). Differences were moderate for Bachelor’s degree vs. High School (*d* = 0.51), and large between Ph.D. vs. Some High School (*d* = 0.80). Further, the ages of peak cognitive performance for each educational category closely followed the typical range of ages at graduation. This result is consistent with a cumulative effect of recent educational experiences, as well as a decrement in performance as completion of schooling becomes more distant. To begin to characterize the directionality of the relationship between educational attainment and cognitive performance, we conducted a prospective longitudinal analysis. For a subset of 69,202 subscribers who had completed 100 days of cognitive training, we tested whether the degree of novel learning was associated with their level of education. Higher educational attainment predicted bigger gains, but the differences were small (*d* = 0.04–0.37). Altogether, these results point to the long-lasting trace of an effect of prior cognitive challenges but suggest that new learning opportunities can reduce performance gaps related to one’s educational history.

## Introduction

Across industrialized nations, only a minority of adults complete post-secondary education. For example, fewer than 40% of adults in the United States are expected to graduate from college in their lifetimes, and the percentage shrinks for more advanced degrees [1]. Considering the cost of higher education, many wonder whether it is a worthwhile investment. Nevertheless, post-secondary educational attainment has been consistently linked to financial and non-monetary benefits [2]. Higher education is intended to confer the qualifications needed for the workforce, but also to improve individuals’ critical thinking and readiness towards life-long learning [3].

Indeed, universities may offer enriching experiences that enhance domain-general abilities to think and learn, such as thinking quickly (processing speed), keeping information in mind (working memory), responding flexibly to task goals (cognitive control), and tackling novel problems (reasoning). Although these skills are not explicitly taught in school, they may serve as a scaffold for learning and have been implicated in academic performance [4,5].

Prior research suggests that education has a positive effect on measures of intelligence [6]. For instance, longitudinal studies using data from compulsory military service in Scandinavian countries have estimated that each completed year of secondary school translates into a gain of nearly two to four IQ points during adolescence [7] and early adulthood [8]. Moreover, the effects of schooling might be strongest for lower-performing individuals [9]. The benefits of schooling have also been shown to be present in old adulthood, such that years of education predict IQ performance at the age of 70, even when controlling for individual differences in IQ at age 11 and other family characteristics such as parental socioeconomic status [10].

Analysis of data collected during periods of significant educational reform has provided even stronger evidence for the causal role of schooling on IQ. The most notable example is data analyzed by Brinch and Galloway [11], which spans nearly two decades when the Norwegian government raised compulsory schooling from seven to 9 years. The authors exploited the fact that different municipalities adopted the reform at various times and that men take a mandatory IQ test on the entrance to military service at age 18. These factors allowed the authors to compare the IQ of individuals who were able to leave school earlier than others. The analysis of this data indicated on average a benefit of nearly four IQ points for each year of schooling.

Aside from the composite measures of IQ used in the longitudinal and quasi-experimental studies described thus far, the effects of schooling have also been reported in studies that examine performance separately on tests that include scholastic knowledge and more abstract tests of cognition. Education positively predicts performance on the subcomponents of a typical IQ test including the reasoning and verbal portion [12,13], and it is a stronger predictor of performance on tests that directly measure skills taught in school, such as math and reading [12]. The length of schooling has also been shown to positively predict performance on a measure of cognitive control in adolescence [14] and measures of reasoning and working memory, but not processing speed, in old adulthood [15]. These findings suggest that educational experience has differential moderating effects on different aspects of cognition.

This prior body of work supports the notion that education positively influences higher cognition, consistent with principles of experience-dependent brain plasticity, from which one would predict improvements in cognitive skills that are repeatedly taxed in demanding and cognitively engaging coursework. However, the scope of prior work limits the conclusions that can be drawn, because they have focused on 1) limited cognitive domains or narrow age groups, 2) are mostly based on Scandinavian men who enlisted in the military, 3) lack the power needed to test the effects of different school levels or adequately characterize the effects of education across the life span, and/or 4) have not examined the impact of education on future learning. Here, we seek to build on prior work by addressing each of these issues.

### The present study

The goal of this study is to better understand the cognitive effects of education by testing whether educational attainment relates to cognitive abilities at one timepoint (a retrospective longitudinal approach), as well as learning efficacy from one timepoint to another (a prospective longitudinal approach). To this end, we examined performance on eight cognitive assessments of executive functioning and reasoning in a diverse sample of over 195,000 individuals, ages 15–60, who had subscribed to an online cognitive training program. Over 69,000 of these subscribers completed these assessments a second time approximately 100 days later, making it possible to evaluate practice-related gains in cognitive performance. We controlled for many variables, including income, sex, ethnicity, native language, and engagement with the training. Importantly, the engagement measures allowed us to quantify and control for individual differences in motivation in our learning context.

We hypothesize that if the cognitive assessments used here capture skills that are relevant to real-world outcomes, we should detect differences in performance associated with educational attainment [6] in addition to age [13,16]. Given previous findings showing positive cognitive outcomes associated with continuing education in adolescence/young adulthood [7,8,11,14], we predicted a significant benefit of completing high school relative to not finishing it, and a further benefit of completing college. We also considered it plausible that there might be differences between holders of graduate degrees relative to college degrees, given differences in the years of higher education required.

Although we sought to understand the influence of education on cognition, it is incontrovertible that cognitive functioning itself influences educational attainment [17]. Some have argued that financial constraints, and not intellectual potential, are the major roadblock in educational attainment [18], but a selection bias is still to be expected, such that students with greater scholastic aptitude are more likely to pursue and attain higher degrees. While we cannot solve this chicken-and-egg problem short of randomly assigning students to pursue different degrees, the analyses described below help to address the question of how, and the degree to which, cognitive performance and learning efficacy vary as a function of prior education.

First, we examined whether educational attainment modulates age-related changes in cognitive performance at one timepoint. Previous work examining changes in cognition through the lifespan has shown that performance on some of the cognitive skills tested here peaks in late adolescence or early adulthood and declines thereafter [16,19]. Given the size and wide age range of our sample, it was possible to test whether these age effects are influenced by education–and, importantly, to determine how the cognitive effects of educational attainment differ across the lifespan, as one’s experience with formal education recedes into the past and is supplanted by other life experiences. To this end, we explored whether educational attainment modulates ages of peak cognitive performance, such that the age of maximal cognitive performance for participants who have achieved a given level of education varies as a function of the age at which this degree is typically completed. We expected to replicate findings showing that late adolescence and early adulthood are the periods during which performance peaks for comparable measures of cognition [16,19]. However, we further hypothesized that maximal cognitive performance would coincide with or closely follow the age at which education was completed. As an initial test of this hypothesis, we compared, for each educational level, the average age of peak cognitive functioning with the average age of graduation.

Second, we examined whether educational attainment differentially modulates performance on the eight individual cognitive assessments. Based on prior work in old adulthood showing the differential effect of schooling on various cognitive measures [15], we predicted that educational attainment would moderate age-related effects on tests of higher-level cognition, such as measures of reasoning, to a greater degree than on tests of lower-level cognition, such as measures of processing speed.

Finally, we examined prospectively whether educational attainment modulates learning efficacy. It has been argued that the effects of education are cumulative, such that quantity of schooling influence the acquisition and maintenance of cognitive skills over time [6,20]. To date, however, there is scant evidence for or against this hypothesis. Here, we sought to test whether prior education modulates practice-related gains in cognitive performance. To this end, we analyzed data from the subset of participants (*n* = 69, 202) who had completed the cognitive assessments before and after engaging with a cognitive training program. We considered three equally plausible outcomes. First, findings in the cognitive training literature [21] raise the possibility that people starting with lower scores would improve the most. Conversely, considering the proposed cumulative effect of education and the well-documented Matthew effect [22], another possibility is that higher levels of education would predict greater gains. Lastly, given that the training games and assessments are unrelated to educational curricula, a third possibility is that there would be no effect of education on the magnitude of practice-related improvements.

To summarize, we examined retrospectively how educational attainment relates to cognitive performance in a large sample spanning the ages of 15–60. We examined the variance captured by educational attainment across the entire age range, and characterized how education moderates performance on individual cognitive assessments and well-established age-related changes in performance (i.e., ages of peak cognitive functioning). Finally, with our prospective analysis, we examined the effect of educational attainment on changes in cognitive performance before and after participation in a cognitive training program that taxes various aspects of executive functioning and reasoning.

## Materials and methods

### Participants

Data were collected from Lumosity subscribers who had answered demographic questions and completed an online battery of cognitive assessments at least once. Subscribers were informed in advance that their data would be used for research purposes should they choose to complete the assessments. All data were de-identified and analyzed in aggregate in accordance with Lumos Labs' Privacy Policy (http://www.lumosity.com/legal/privacy_policy).

To be included in this study, participants had to be between the ages of 15–60 and reside in the United States (*n* = 152,694), Canada (*n* = 21,767), or Australia (*n* = 21,927). These countries were selected for several reasons. First, they were the most represented countries in the sample. Second, they share the same official language. Third, education is typically compulsory until the age of 16. Finally, their university systems require equivalent qualifications for admissions into college and graduate programs.

Additionally, we only included participants whose reported age was greater than their years of education (i.e., age > years of education + 4), and whose educational attainment was plausible given both their age and years of schooling (i.e., excluded participants younger than 17 with a Bachelor’s degree or younger than 20 with a graduate degree). Moreover, we recoded participants’ educational attainment as “Associate’s/Some College” if they reported having a Bachelor’s degree but were younger than 20 years old and had less than 15 years of formal education, since their response most likely reflects a clerical error rather than their actual educational attainment (*n* = 181). We chose these cutoffs based on pertinent international statistics of typical graduation ages [1]. Finally, we also included in our analyses data from participants who did not specify their educational attainment level, so as not to bias the normalization procedures of the cognitive assessments scores.

**Fig 1:**
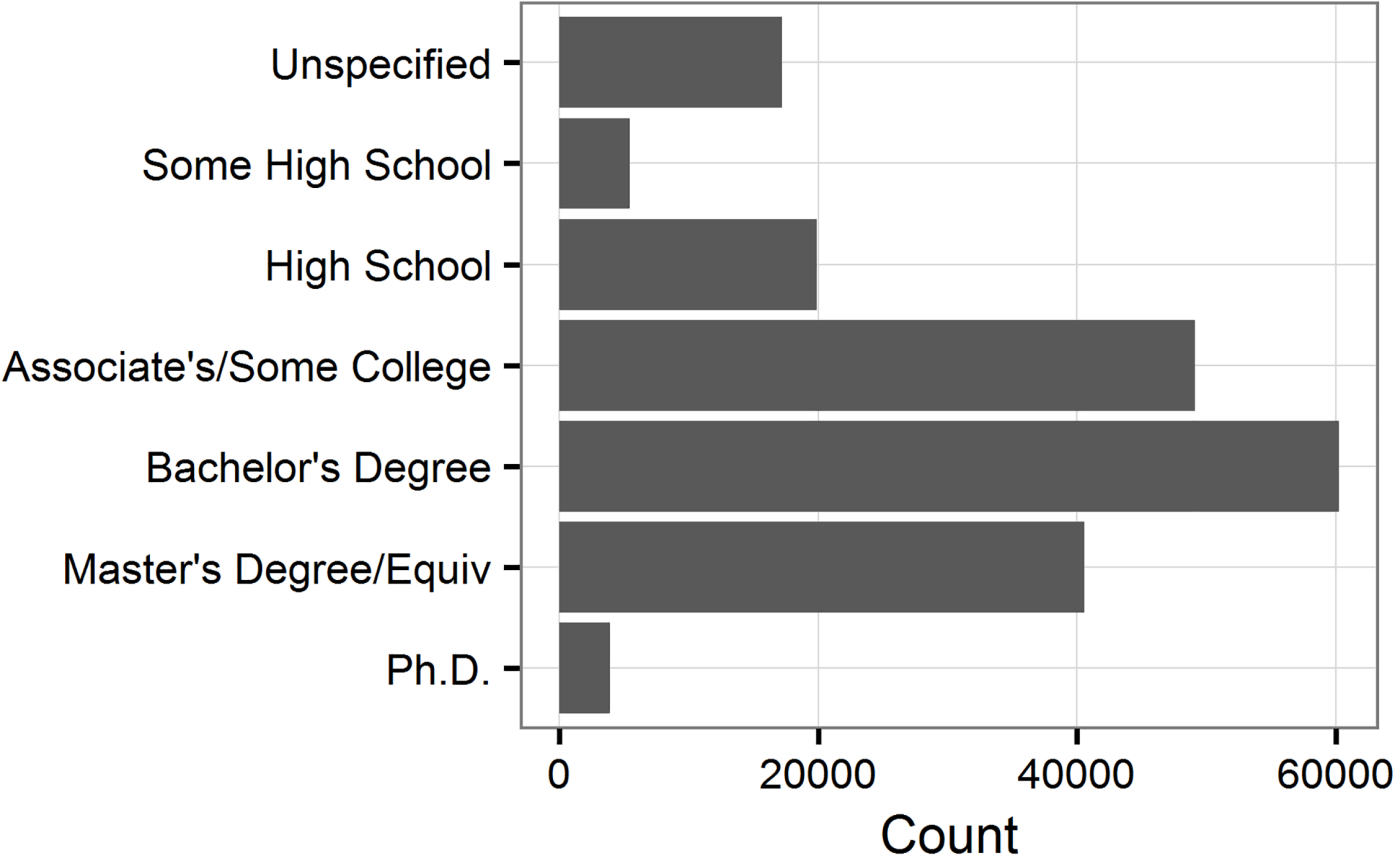
Distribution of educational attainment. The number of participants reporting educational attainment between Some High School to Ph.D.’s at T1 (*N* = 196,388).

Thus, the retrospective single timepoint analyses included 196,388 participants (53.72% females; *M*_age_ = 39.95 ±12.8 *SD*). The prospective learning analyses included a subset of these participants (*n* = 69,202; 58.84% females; *M*_age_ = 43.11 ±12.23 *SD*). The participants were from diverse demographic backgrounds and with educational attainment ranging from some high school to doctoral degrees (Fig 1, skewness _years of education_ = -0.23). Our sample is slightly skewed towards higher educational attainment given that 52% of our sample has attained at least a Bachelor’s degree, vs. ~34% of adults in the countries included in our analysis [1]. However, the distributions of education categories across the age (S1 Fig), income levels (S2 Fig), and ethnic categories (S3 Fig) are consistent with patterns seen in these general populations [1,23].

In addition to establishing education categories, we subdivided our participant into five-year age bins (e.g., 30–35) to maximize our ability to compare similarly represented educational attainment levels and age groups. We subdivided the age range into one-year bins to test whether the age of peak cognitive performance varies as a function of educational history.

### Cognitive assessments

Participants completed a battery of eight assessments designed to evaluate working memory, flexibility, processing speed, and verbal and non-verbal reasoning. The assessments are a valid and reliable [24] computerized adaptation of classic pencil-paper neuropsychological tests accessible online that can be completed in 30 minutes. These tests, including their performance metrics, have been described in detail elsewhere [24]. The working memory tests require participants to hold in mind strings of spatial locations (Forward and Reverse Spatial Span). The processing speed tasks involve quickly connecting numbers in a sequence (Trail Making A) and matching numbers to symbols (Digit Symbol Coding). The test of cognitive flexibility includes connecting in order-interleaved numbers and letters (Trail Making B). Finally, the tests of reasoning involve answering questions about simple logical statements (Grammatical Reasoning), performing simple word-based additions and subtractions (Arithmetic Reasoning), and completing a visual pattern based on rules and relationship between items (Progressive Matrices).

Participants completed these assessments at two timepoints, before (T1) and after (T2) engaging with the cognitive training program. We used T1 data for our retrospective analyses and the change score data (T2-T1) for our prospective analyses.

#### Performance scores and analysis

The cognitive assessments have different performance metrics and distributions. Thus, we standardized the raw scores according to a conventional normalization procedure so that the performance scores will have a normal distribution with mean 100 (15 *SD*). Specifically, raw scores for a given subtest from T1 were ranked and then converted into percentile scores according to the empirical cumulative distribution. Normed scores were created by converting the percentile scores to their corresponding position on a normal distribution (*M* = 100, *SD* = 15). We performed a similar normalization procedure for T2 scores, but they were converted to percentiles according to the empirical cumulative distribution of T1 raw scores to preserve changes in performance from T1 to T2. We used this normalization procedure to generate age-specific normative data.

We created an aggregate measure of T1 and T2 cognitive performance for each participant–a Grand Index (GI) score–by summing together for each timepoint the norm scores of each assessment and normalizing the sum scores to have a distribution with a mean of 100 (15 *SD*). Before testing for cognitive effects of educational attainment, we regressed out from all normed performance metrics the effect of demographic variables and engagement with the Lumosity games. We calculated GI change scores by subtracting the raw GI scores of T1 from T2. Before testing the effects of educational attainment, we regressed out from the change score demographic and engagement variables, as well as the raw T1 score.

Demographic covariates included participants’ reported gender, ethnicity, and whether or not English was their native language. We included gender and ethnicity as covariates to control for differences in access to education and any effects of stereotype threat on cognitive test-taking ability [25]. We also assessed effects of household income, given that income could influence access to education and other cognitively engaging activities. The engagement covariates were the number of hours each participant played the cognitive training games before each assessment and the number of days that elapsed between timepoints. We used the latter covariate only in the calculation of the change score. In all analyses, we used the log form of these engagement variables given their distribution and relationship to cognitive performance. Together, all these covariates accounted for a small variance in cognitive performance at both timepoints (*R*^2^ ≤ 0.05, *p* < 0.0001; S1 Table).

All analyses use the adjusted normed scores resulting from these normalization procedures. The linear models used to probe the relationship of educational attainment with cognitive performance and learning are described in detail in the results section.

#### Peak analysis procedures

To test whether educational attainment modulates the ages of peak cognitive performance, we adopted a bootstrap resampling procedure similar to one previously employed with large cross-sectional online samples [16]. Specifically, we drew a sample between ages of 15–60 (in one-year bins) from each educational category and identified the age group with the highest T1 GI score, using the age-specific normative data and adjusting for all the aforementioned control variables. We repeated the procedure 10,000 times, which allowed us to calculate a median age of maximal performance and the corresponding 95% confidence intervals (CIs). The sample size selected (with replacements) from each education group at each iteration equaled the sample size available for that education category (Fig 1), and we only considered age bins with at least 50 participants for each education level.

To explore the possibility that age at peak cognitive functioning is related to the age at which education is terminated, we accessed international indicators of typical ages of graduation for each educational attainment category [1]. The report listed ages of graduation as a range separately for each country included in our sample, which we used to calculate the median age and typical age ranges of graduation for each education category. For instance, we estimated that for a Bachelor’s degree, the typical ages of graduation are 20 to 24 and the median age is 22.5 given the typical ages of graduation in each country: 21–23 in the USA, 22–24 in Canada, and 20–23 in Australia.

#### Dataset

The R code (v. 3.2.4) and dataset used in all the analyses are available at Open Science Framework https://osf.io/x7x6w/ (DOI 10.17605/OSF.IO/V6K3J).

## Results

### Effects of educational attainment and age on cognitive performance

To test whether educational history modulated cognitive performance across a range of task demands, we used the aggregate measure based on all the cognitive assessments for each participant at T1, termed the Grand Index (GI) score. As described above, we controlled for demographic variables and level of prior engagement with the Lumosity training games.

**Fig 2:**
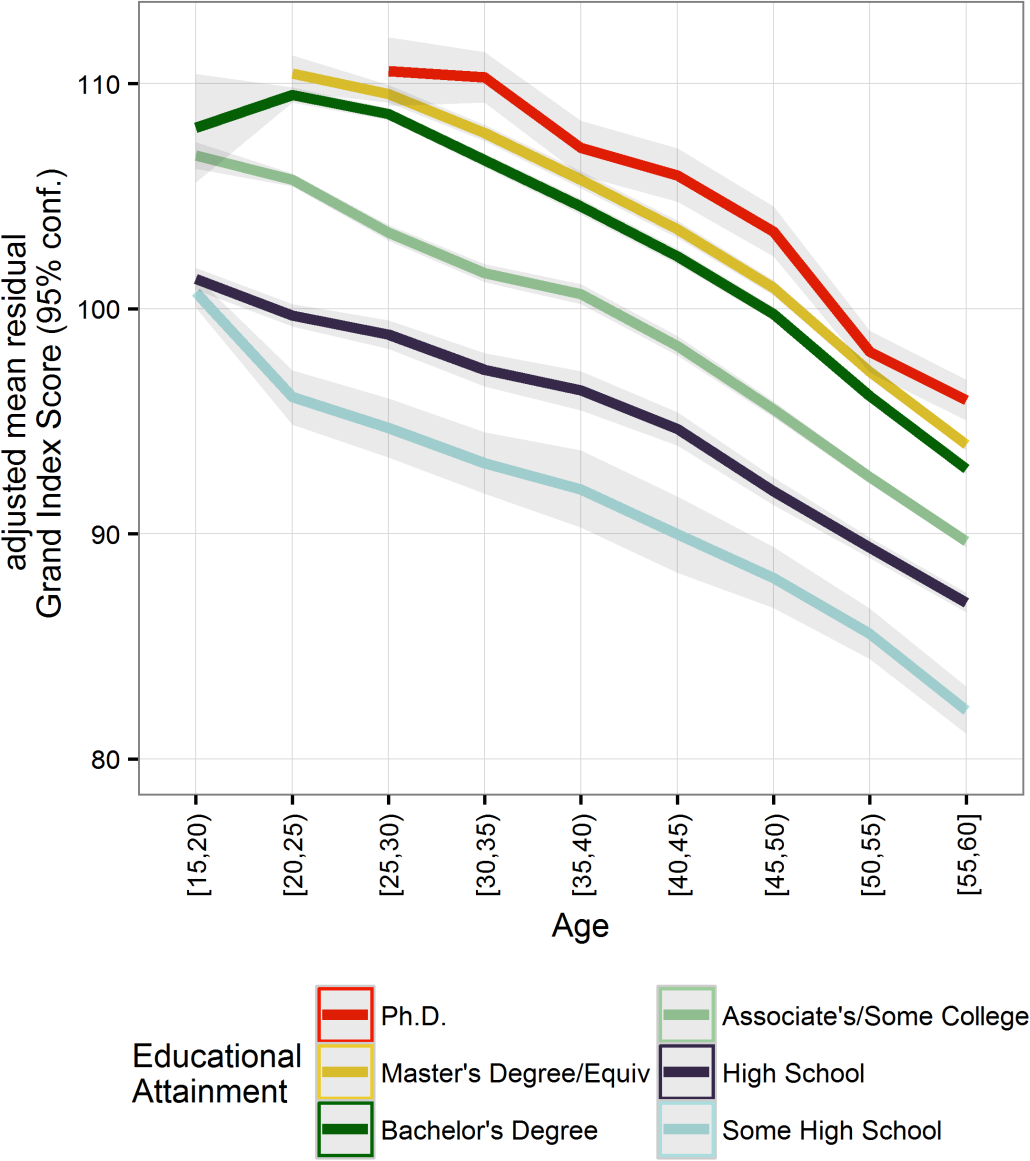
Effect of educational attainment on the adjusted Grand Index score at T1 across the ages of 15 and 60. Ribbons show bootstrapped 95% CIs from 10,000 iterations. Displaying age/education categories with 50+ participants who specified their educational attainment level (*n* = 179,141; *N* = 196,388).

As predicted, educational attainment levels (Some High School, High School, Some College/Associate’s, Bachelor’s, Master’s, Ph.D.) and age (categorical 5-year age bins between ages 15 and 60), were significant predictors of the *adjusted* GI score at T1 (*adjusted R*^2^ = 0.18; *p* < 0.0001; Table 1; Fig 2). Household income did not account for additional variance (*adjusted R*^2^ = 0.18, *p* < 0.0001; Table 1) and was therefore not included in subsequent analysis.

**Table 1:** Educational attainment and age predicting T1 cognitive performance and learning, as measured by the Grand Index (GI) score.

	GI T1 Estimate	Controlling for income GI T1 Estimate	Δ GI Estimate	Controlling for T1 scores Δ GI Estimate	Age regressed out	
					GI T1 Estimate	Δ GI Estimate
**Intercept**	100.63***	100.87***	5.12**	5.20 ***	95.11 ***	2.95***
**Some high school**	-2.73***	-2.69***	0.05	-0.33	-1.80 ***	-0.31
**Assoc./Some College**	3.90***	3.90***	0.31**	0.69***	3.10 ***	0.73***
**Bachelor’s**	7.83***	7.78***	0.42***	1.20 ***	6.63 ***	1.31***
**Master’s/equivalent**	8.91***	8.83***	0.52***	1.42 ***	7.65 ***	1.58***
**Ph.D.**	10.81***	10.74***	1.12***	2.20 ***	9.64 ***	2.44***
**Unspecified**	3.43***	3.46***	0.36*	0.78***	2.50 ***	0.76***
**Ages 20–25**	0.52**	0.39**	-0.43*	-0.27		
**Ages 25–30**	-0.39*	-0.58*	-0.70***	-0.63**		
**Ages 30–35**	-2.14***	-2.36***	-0.98***	-1.05 ***		
**Ages 35–40**	-3.86***	-4.10***	-1.42***	-1.67 ***		
**Ages 40–45**	-5.97***	-6.22***	-1.81***	-2.27 ***		
**Ages 45–50**	-8.59***	-8.85***	-1.73***	-2.49 ***		
**Ages 50–55**	-11.99***	-12.23***	-1.73***	-2.86 ***		
**Ages 55–60**	-15.11***	-15.33***	-2.07***	-3.51 ***		

The GI change score (ΔGI) is the difference between the GI score from T1 and T2. Each GI score was normalized to have a distribution with mean of 100 (15 SD) and was adjusted for the effects of demographic covariates (gender, ethnicity, and indicator of English as native language), engagement variables (number of gameplay hours and days between T1 and T2), T1 performance (T2 only), and other specified variable. The two rightmost columns additionally regress out the effects of age (5-age bins). Reference category: ages 15–20 and High School attainment. *p* < 0.0001‘***’, *p* < 0.001‘**’, *p* < 0.01‘*’

#### Quantifying the unique influence of educational attainment

Having found that GI scores vary as a function of both age and education, we sought to quantify the amount of unique variance explained by the latter. To this end, we regressed out the 5-year age bins in addition to the demographic and engagement covariates when calculating the GI score. A linear regression showed that educational attainment positively predicted a significant, albeit small, amount of variance in cognitive performance (*adjusted R*^2^ = 0.04, *p* < 0.0001). All educational attainment categories were significant predictors of the GI score at T1, showing a pattern of results in line with the additional years of schooling required to complete each degree (Table 1).

Next, we sought to more carefully characterize the effects of different levels of educational attainment. Thus, we calculated pairwise differences in effect size between each educational attainment levels using Cohen’s d (Table 2). Differences in effect sizes were relatively large between the extremes of educational attainment (Ph.D. vs. Some High School, *d* = 0.80), moderate at a key educational juncture (Bachelor’s vs. High School, *d* = 0.51), and small between other adjacent education levels (e.g., Master's vs. Bachelor's, *d* = 0.08).

**Table 2:** Pairwise differences in effect size between educational attainment levels at T1.

	Some High School	High School	Some College/ Associates	Bachelor's	Master's or equivalent
**High School**	0.13 *	-			
**Some College/ Associates**	0.36 *	0.23 *	-		
**Bachelor's**	0.65 *	0.51 *	0.27 *	-	
**Master's or equivalent**	0.72 *	0.59 *	0.35 *	0.08 *	-
**Ph.D.**	0.80 *	0.71 *	0.49 *	0.23 *	0.16 *

Pairwise effect sizes (Cohen’s d) were calculated between each educational attainment category predicting T1 Grand Index Score with age regressed out. CIs calculated using 10,000 bootstrap iterations.

‘*’ 95% CIs does not include 0.

#### Educational attainment moderates the age of peak cognitive performance

**Fig 3:**
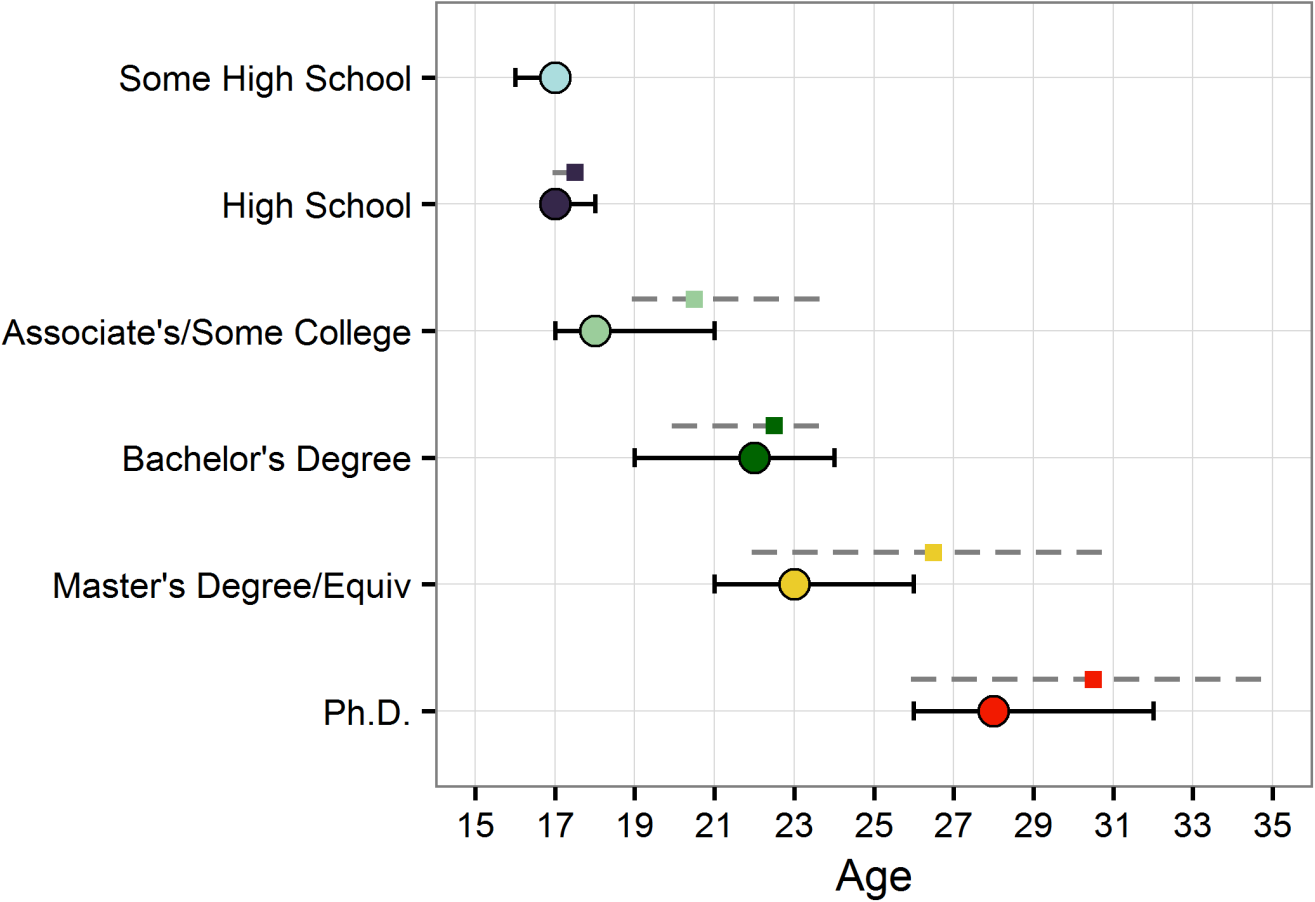
Educational attainment moderates the ages when cognitive performance peaks. Colored points show the median age of maximum performance and the error bars the 95% CIs. We calculated these ages using a 10,000 iteration bootstrap sampling procedure including the entire age range available in our sample (ages 15–60). Gray dotted lines represent the age range, and the colored squares represent the median age of typical graduation for each education level. We obtained these graduation ages from international indicator reports, which included data from all three countries represented in our sample (USA, Canada, and Australia).

Having found that education and age are stronger predictors of performance than education alone, we performed a granular analysis examining the effects of educational attainment on cognitive performance across the lifespan. Specifically, we tested whether the age of maximum performance differed between education categories. The literature suggests that late adolescence/early adulthood is when performance peaks on comparable measures of cognition [16,19]. We reasoned that if educational attainment influences cognitive performance and these effects are greater as the educational experience are more recent, the age of peak performance would vary as a function of education and be proximal to the ages when people typically complete the education programs. We found that ages of peak performance were within the young adulthood period, occurred later the higher the education level, and overlapped with typical ages of graduation for each degree (Fig 3). Specifically, 17 was when performance was maximum for the High School and age 22 for the Bachelor’s category. These ages were well-aligned with the typical ages of graduation from those educational categories, 17.5 and 22.5 respectively [1]. Ages of peak performance for the other degrees were within the range of typical ages of graduation for their respective program. Importantly, peak performance was not at the youngest possible ages for each education category, which suggests that we may not be just capturing an effect of age or performance from higher achieving individuals (i.e., youngest people to receive a postsecondary degree), but instead the effects of a recent educational experience.

#### Influence of educational attainment on individual cognitive measures

Thus far, we have presented how education relates to cognitive performance on an aggregate measure (GI) because it is the most reliable and robust measure [24]. However, we also sought to explore the possibility that educational attainment was specifically or preferentially related to a subset of the eight cognitive assessments, moderating typical age-related changes in performance. Thus, we tested the effect of educational attainment and age on performance on tests of working memory, processing speed, cognitive flexibility, and verbal and non-verbal reasoning. The tests were free of educational content–except for Arithmetic Reasoning, which involved elementary scholastic content (e.g., simple word-based additions and subtractions)—that all our participants are expected to have been exposed to in school. We hypothesized that higher education would have its largest effect on measures of reasoning, given the complex, abstract material covered in college and beyond, and that it would have its smallest effect on tests of processing speed.

**Fig 4:**
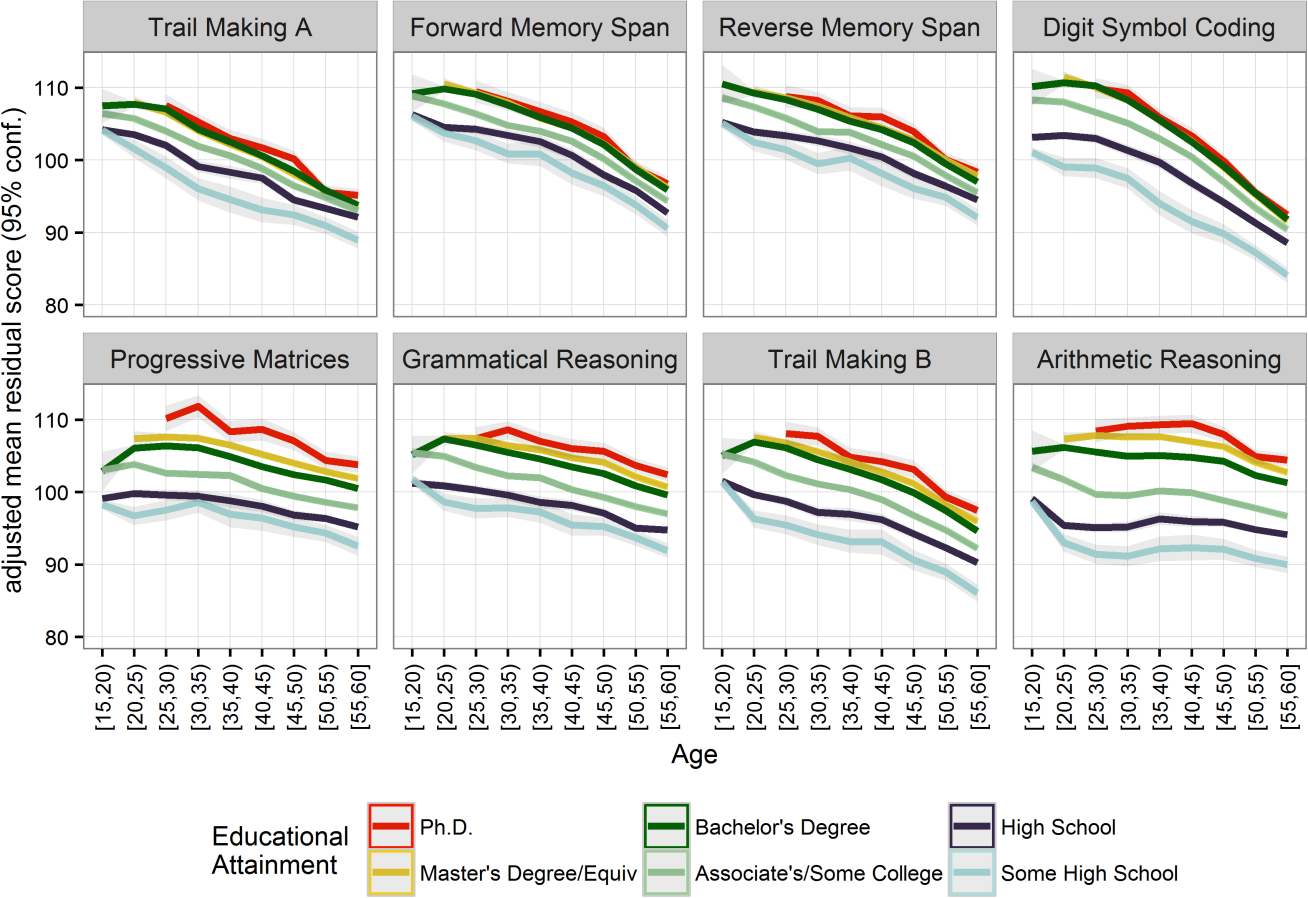
Effects of educational attainment on individual cognitive assessments across ages 15–60 (*n* = 179,141). Ribbons show bootstrapped 95% CIs based on 10,000 iterations. Displaying age/education categories with 50+ participants who indicated an educational attainment level (*N* = 196,388).

As predicted, age and education predicted distinct amounts of variance in performance on different assessments (Fig 4). On simple tests like Trail Making A, which requires speeded responding based on a simple rule, there was a noticeable age-related decline and a weaker effect of education. By contrast, scores on more cognitively complex tests, including Arithmetic Reasoning, Grammatical Reasoning, and Progressive Matrices, showed greater variance explained by educational attainment when controlling for age (S4 Fig). These assessments also tended to show later peak performance and initial points of decline as a function of age.

Considering that the test that was most strongly modulated by education, Arithmetic Reasoning, required numerical competencies (albeit skills taught in elementary school), we tested whether our results would hold when excluding this assessment. Indeed, excluding it from the calculation of the GI score at T1 did not impact the amount of variance educational attainment explained, nor the differences in effect sizes between education levels. For example, educational attainment and age (categorical 5-year age bins between ages 15 and 60) remained significant predictors of the adjusted GI score (*adjusted R*^2^ = 0.19; *p* < 0.0001). Thus, educational history modulated performance even on tasks that bore no resemblance to those encountered in the classroom.

### Effects of educational attainment and age on learning

To test whether prior educational attainment influences how quickly one learns, we took advantage of the fact that 69,202 of the subscribers in the sample took the cognitive assessments twice, on average 100.77 days (± 57.67 *SD*) apart, playing a suite of cognitive games in the interim (*M* = 166.23 hours ± 290.20h *SD*). These games were different from the assessments but were designed to tax the same underlying cognitive skills [26]. The goal of this study was not to assess the overall effectiveness of the training [26], but rather to test whether educational attainment would be associated with the magnitude of gain in the GI score. Thus, we calculated a GI change score as the difference in performance between the assessments taken after (T2) and before the training program (T1). We then tested whether educational attainment modulated the GI change score, regressing out the effects of the same demographic variables as T1, engagement in the training program (i.e., number of hours of gameplay and days elapsed between assessments), and T1 performance (to control for the plausible effect of regression to the mean).

**Fig 5:**
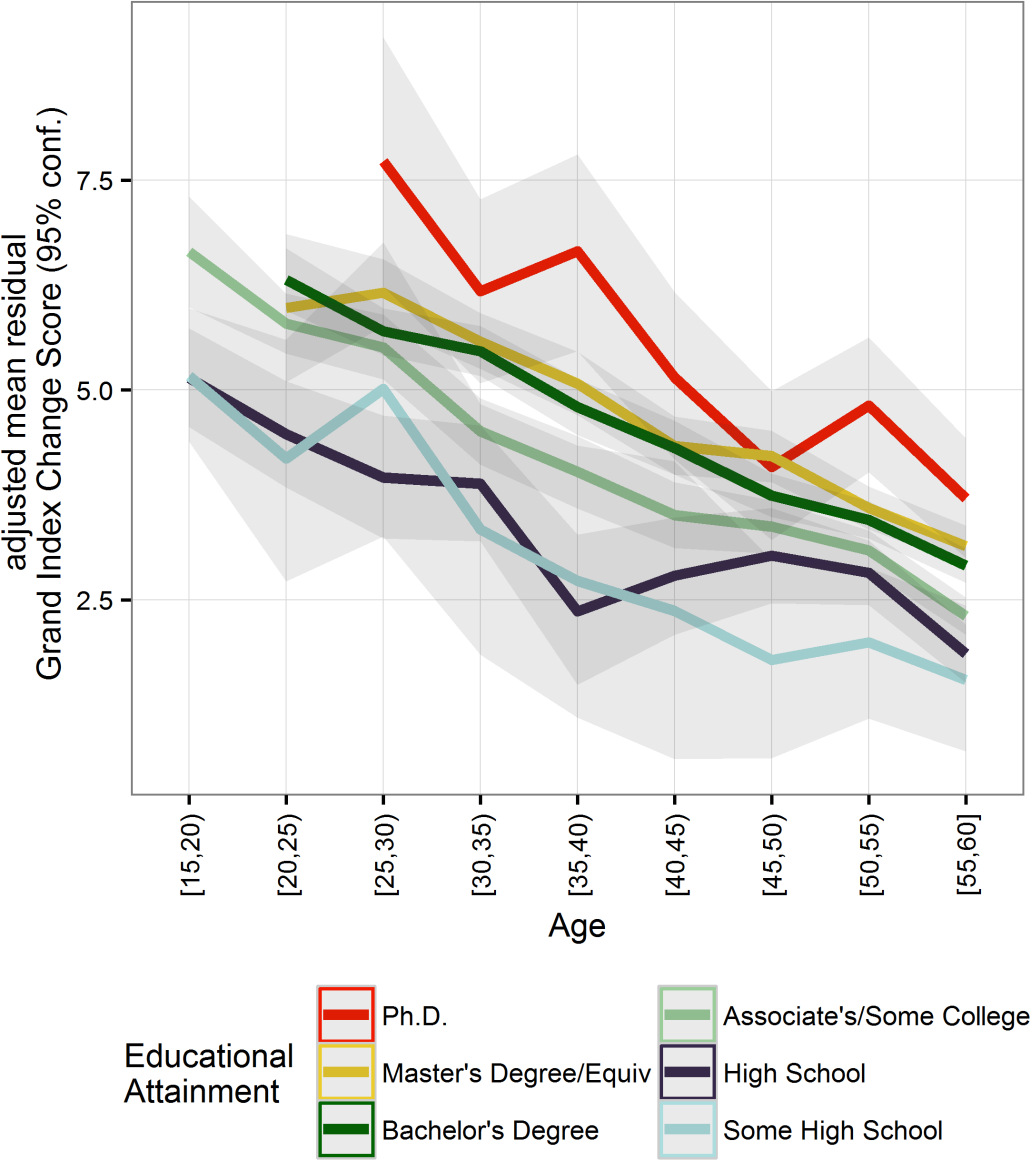
Effect of educational attainment on adjusted GI change score across ages 15–65 (*n* = 63,535). Y-axis units represent the change in GI scores from T1 to T2. At each timepoint, the GI scores were normalized to have a distribution with a mean of 100 (15 *SD*). Ribbons show bootstrapped 95% CIs based on 10,000 iterations. Displaying age/education categories with 50+ participants who indicated an educational attainment level (*N* = 69,202).

We found a minimal effect of educational attainment on training-related gains on the adjusted GI change score (*adjusted R*^2^ = 0.03, *p* < 0.0001; Table 1; Fig 5). This effect held whether or not we controlled for T1 performance (without controlling for T1: *adjusted R*^*2*^ = 0.01, *p* < 0.0001; Table 1). Post-secondary educational attainment predicted larger improvements, ranging from 0.5 to 2 points higher relative to the High School 15–20 age group reference category (*b*_*0*_ = 5.20, *p* < 0.0001). There was also a general effect of age, wherein age groups younger than 30 showed larger gains from training (e.g., *b*_*25-30 HS*_ = -0.63, *p* < 0.0001) than the older groups (e.g., *b*_*55-60* HS_ = -3.51, *p* < 0.0001). Thus, higher educational attainment and youth both predicted higher learning efficacy.

#### Quantifying the unique influence of educational attainment on learning

We next sought to quantify the effect of educational attainment on the change in cognitive performance independently of the effect of age. To this end, we calculated the GI change score with the 5-year age bins as additional covariates, following similar procedures to the analogous T1 analysis. Educational attainment accounted for a negligible amount of variance in the adjusted GI change score (*adjusted R*^*2*^ = 0.01, *p* < 0.0001; Table 1). Post-secondary attainment predicted additional improvements from the High School reference category, but overall, participants across education levels exhibited similar gains. The fact that educational attainment was not negatively related to the change score leads us to reject the hypothesis that individuals that are more educated stood to gain less from practice than their peers because of their higher starting performance level.

We also compared the effects of different levels of educational attainment with pairwise differences in effect size between each education category (Table 3). As with T1 data, differences in effect sizes for the magnitude of learning were larger between the extremes of educational attainment (Ph.D. vs. Some High School, *d* = 0.37) than between adjacent levels (e.g., Master's vs. Bachelor's, *d* = 0.04). All of the results above of the effects of educational attainment on learning were replicated after excluding the Arithmetic Reasoning test from the calculation of the GI score at both timepoints. Thus, as with cognitive performance at one timepoint, practice-related gains were associated with educational attainment even for non-academically related tasks.

**Table 3:** Pairwise differences in effect size between educational attainment levels in the change score analysis.

	Some High School	High School	Some College/ Associates	Bachelor's	Master's or equivalent
**High School**	0.04	-			
**Some College/ Associates**	0.14 *	0.10 *	-		
**Bachelor's**	0.22 *	0.18 *	0.08 *	-	
**Master's or equivalent**	0.26 *	0.22 *	0.12 *	0.04 *	-
**Ph.D.**	0.37 *	0.33 *	0.24 *	0.16 *	0.12 *

Pairwise effect sizes (Cohen’s d) were calculated between each educational attainment category predicting Grand Index Change Score with age regressed out. CIs calculated using 10,000 bootstrap iterations.

‘*’ 95% CIs does not include 0.

## Discussion

We sought to test the extent to which cognitive performance and learning efficacy in adolescence and adulthood vary as a function of educational attainment, ranging from some high school to advanced degrees. To this end, we analyzed data from a large sample of 15-60-year-olds who completed eight cognitive assessments. Controlling for multiple demographic and engagement variables, we found that educational attainment had a small but significant overall effect on performance at the initial timepoint, but had a negligible effect on learning efficacy. There were moderate differences in performance between secondary and post-secondary education levels, but minimal ones between post-secondary levels.

Peak analyses revealed that the higher the education level, the later the age of maximal cognitive functioning. Further, the peaks overlapped with typical graduation ages for the different degrees. This overlap was tighter for the High School and Bachelor’s degree, for which the age of maximal performance coincided almost exactly with typical ages of graduation. For the other degrees, the age of peak performance was within the range of typical graduation ages. These results suggest that we may be capturing the cumulative effect of a recent educational experience. Although these preliminary results are cross-sectional and the dataset did not include the age at which individual participants completed their education, they are suggestive of an age-related cognitive decline beginning shortly thereafter.

The variance in performance explained by education was greater in some cognitive domains, such as reasoning, but nonetheless smaller compared to the effects of age. However, the effect of education was present across the broad age range, persisting for decades beyond typical graduation ages. Although we did not have a principled way of rank-ordering or grouping the eight assessments according to their level of cognitive complexity, our results are suggestive of the idea that education affects higher-level cognitive functions more strongly than lower-level ones. This observation complements previous findings [15] by documenting the effect across the five decades of life spanned by our sample.

We found a modest effect of educational attainment on learning, as indexed by gains on the cognitive assessments after completing the training program. Post-secondary education categories exhibited only slightly larger gains than secondary levels. Moreover, High School graduates reached scores at post-test that were comparable to those attained at pre-test by individuals who had completed some college. Thus, practice may reduce gaps in performance observed as a function of educational history. The fact that education had only a small effect on learning is perhaps not surprising, given that the training program was not academic in nature. The cumulative benefits of education may be more salient when the curricula build directly on academic knowledge and skills explicitly taught in school.

### Limitations and future directions

Our study has several limitations. First, it is impossible with our cross-sectional dataset–or even with our dataset including two timepoints of data per individual–to prove conclusively that higher education hones domain-general cognitive functioning. In fact, conclusively teasing apart the bi-directional influence of cognition and education is insurmountable even when individuals are followed for years because having an initial assessment of cognitive performance before undergoing an educational experience does not preclude the effect of other confounding variables (e.g., motivation) [9]. Data from “natural experiments,” such as instances of school reform, can provide stronger evidence on the directionality of effects, but these situations are rare and may suffer from other confounding variables [11].

Despite this inherent challenge, the current study complements those that have employed a longitudinal design or a naturalistic experiment, since we demonstrate a positive relationship between educational attainment and cognitive performance within the range of effects in literature. Moreover, the unique size and heterogeneity of our sample allow us to quantify the effects of educational attainment relative to other factors, including the influence of demographic and engagement variables. Further, our analysis of age at peak cognitive functioning suggests that we are able to capture the effects of recent educational experiences on cognition and not just general effects of age. Also, the fact that our cognitive tests are so different from school curricula yet show differential effects of education helps further disambiguate the relationship between educational attainment and cognitive performance in our study. Finally, the test-retest data allowed us to test how prior educational experiences influence the efficacy of new learning.

A second limitation is that our analyses hinged on self-reports of age, education, income, and other demographic variables. Errors in self-reporting could have led to miscategorization of participants, even though we took steps to remove participants whose answers were incongruous. If anything, however, miscategorization would introduce noise into the dataset that would likely lead to an underestimation of the effect sizes. We also do not have a measure of the participant’s childhood socioeconomic status or whether they completed their education in the countries included. Again here, however, such errors should reduce rather than inflate our ability to detect predicted effects. Relatedly, not knowing when our subjects completed their degrees, but instead inferring this information from reports of international indicators, constrains the interpretations that can be drawn from our analysis examining whether educational attainment modulates ages of peak cognitive performance. However, to maximize the possibility that our sample was representative of the larger population, we only included in the analysis age groups with a representative number of subjects in each education category.

Another potential limitation is that there is a possible selection bias for subscribing in Lumosity. However, the broad distribution of our sample’s education across demographic variables is similar to patterns observed in the general population, and the age-related declines in performance are systematic and consistent with the literature. We could alternatively consider this potential limitation as a feature of the dataset. If there is, in fact, a selection bias for subscribing to Lumosity such that our sample represents individuals who are motivated to pursue cognitive enriching activities despite their educational history, then motivation-related confounds–that have been hypothesized to drive in part the relationship between education and cognitive performance [9]–should be attenuated in this sample.

Finally, facility with computers could have contributed to our findings, given that the assessments were computerized and that prior experience with computers likely varied both as a function of age and education [1]. However, our results are inconsistent with this account: for one, individual assessments were modulated differently by age or education; for another, if the results reflected computer skills or acquisition, the subset of participants expected to have better computer skills (e.g. higher education/younger ages) should benefit the least from the training program, not the most.

Our unique dataset allowed us to begin answering important questions about the cognitive effects of education that should be further investigated with longitudinal studies. For instance, performance differences between education levels were evident from our earliest 5-year age bin group. However, it remains an open question whether the magnitude of the education effects increase, narrow, or remain stable with age. Additionally, findings from the peak analysis showing that maximal performance and the subsequent decline occurs later in the higher education levels are consistent with the idea that higher education may help to stave off age-related cognitive decrements [20, 27]. These observations raise a question about whether or how the timing of the educational experiences impacts cognitive functioning over the long term. Finally, our results from the learning efficacy analysis are consistent with findings showing that young adults show larger gains than older adults from cognitive interventions [28]. The question remains, however, the degree to which prior educational experiences interact with the effects of age. The answers to these and similar questions have theorerical implications to increase our understanding of the sensitive periods in the development of higher cognition and its plasticity through adulthood, as well as practical implications for govermental decisions about school reform and policy geared towards increasing inclusive access to and completion of higher education.

In conclusion, our results indicate a relation between educational attainment and cognitive abilities across a broad age range but small effects on learning efficacy. Although our results are statistically significant, even after controlling for multiple potential confounds, the amount of variance explained by educational attainment is small. In a smaller-scale study, these effects would probably not have been detected at all [5]. Nonetheless, these findings support the idea that higher education provides the opportunity to hone domain-general cognitive skills as well as to acquire content knowledge and that education-related gap in performance can be mitigated with intensive cognitive engagement.

## Supporting information

S1 Fig Distribution of educational attainment across age groups. The number of participants between the ages of 15–60, reporting educational attainment between Some High School to Ph.D.’s at T1 (*N* = 196,388).
(TIFF)

S2 Fig Distribution of educational attainment across household income. The number of participants who identified their household income bracket in U.S. dollars, and reported their educational attainment between Some High School to Ph.D.’s at T1 (*N* = 196,388).
(TIFF)

S3 Fig Distribution of educational attainment across ethnicity categories. The number of participants across ethnic categories, reporting educational attainment between Some High School to Ph.D.’s at T1 (*N* = 196,388).
(TIFF)

S4 Fig Unique variance in cognitive performance explained by educational attainment at T1. Displaying *R*^*2*^ values from regression models of educational attainment predicting performance on individual cognitive assessments.
(TIFF)

S1 Table Demographic and engagement covariates predicting T1 cognitive performance and learning, as measured by the Grand Index score (GI). (PDF)
