# Supplementary figures and images for: Does higher education hone cognitive functioning and learning efficacy? Findings from a large and diverse sample
Source: PLoS One. 2017 Aug 23;12(8):e0182276. doi: 10.1371/journal.pone.0182276 (PMC5568102; doi:10.1371/journal.pone.0182276)

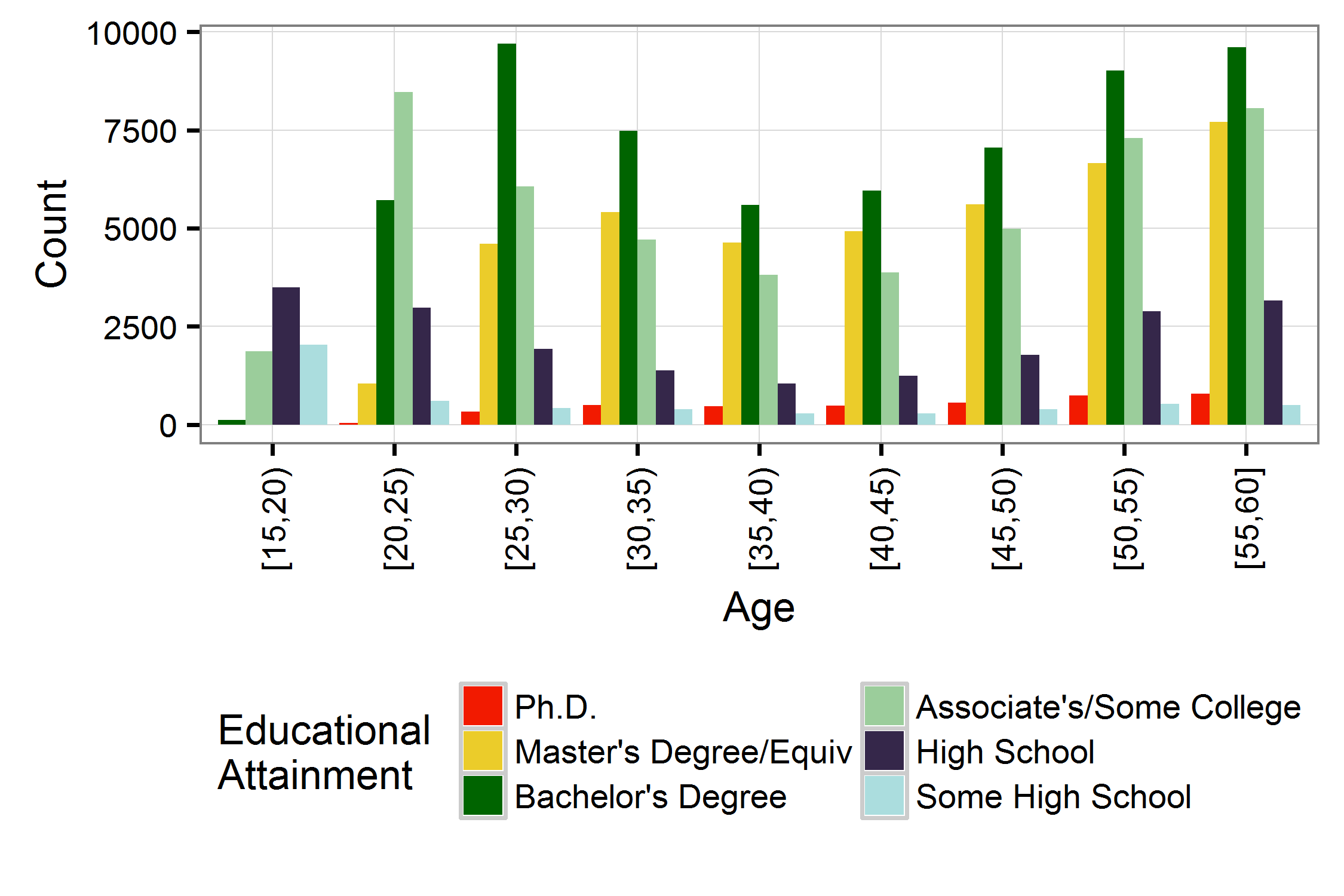

Supplement: S1 Fig — The number of participants between the ages of 15–60, reporting educational attainment between Some High School to Ph.D.’s at T1 (N = 196,388). (TIFF) [file pone.0182276.s001.tiff]

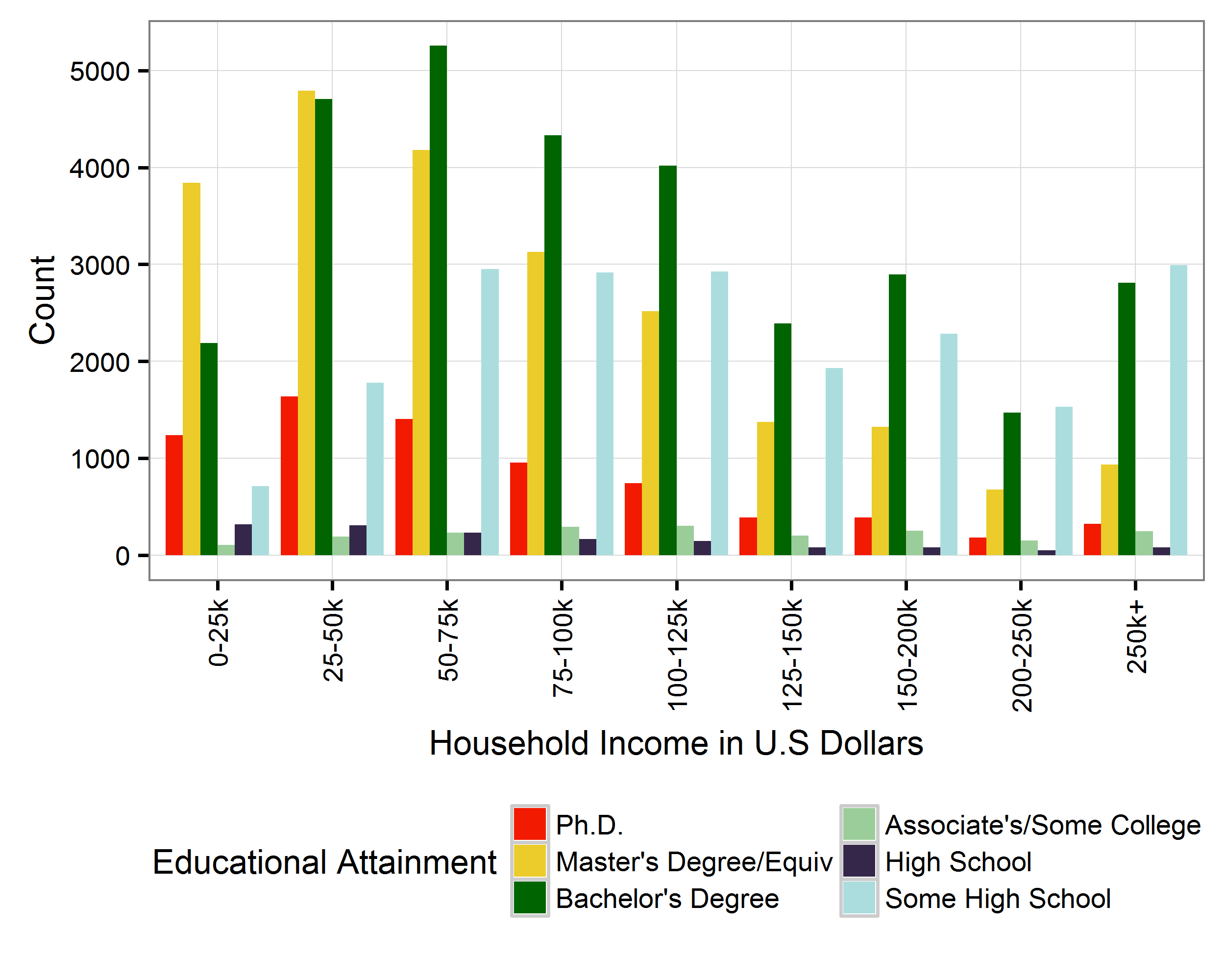

Supplement: S2 Fig — The number of participants who identified their household income bracket in U.S. dollars, and reported their educational attainment between Some High School to Ph.D.’s at T1 (N = 196,388). (TIFF) [file pone.0182276.s002.tiff]

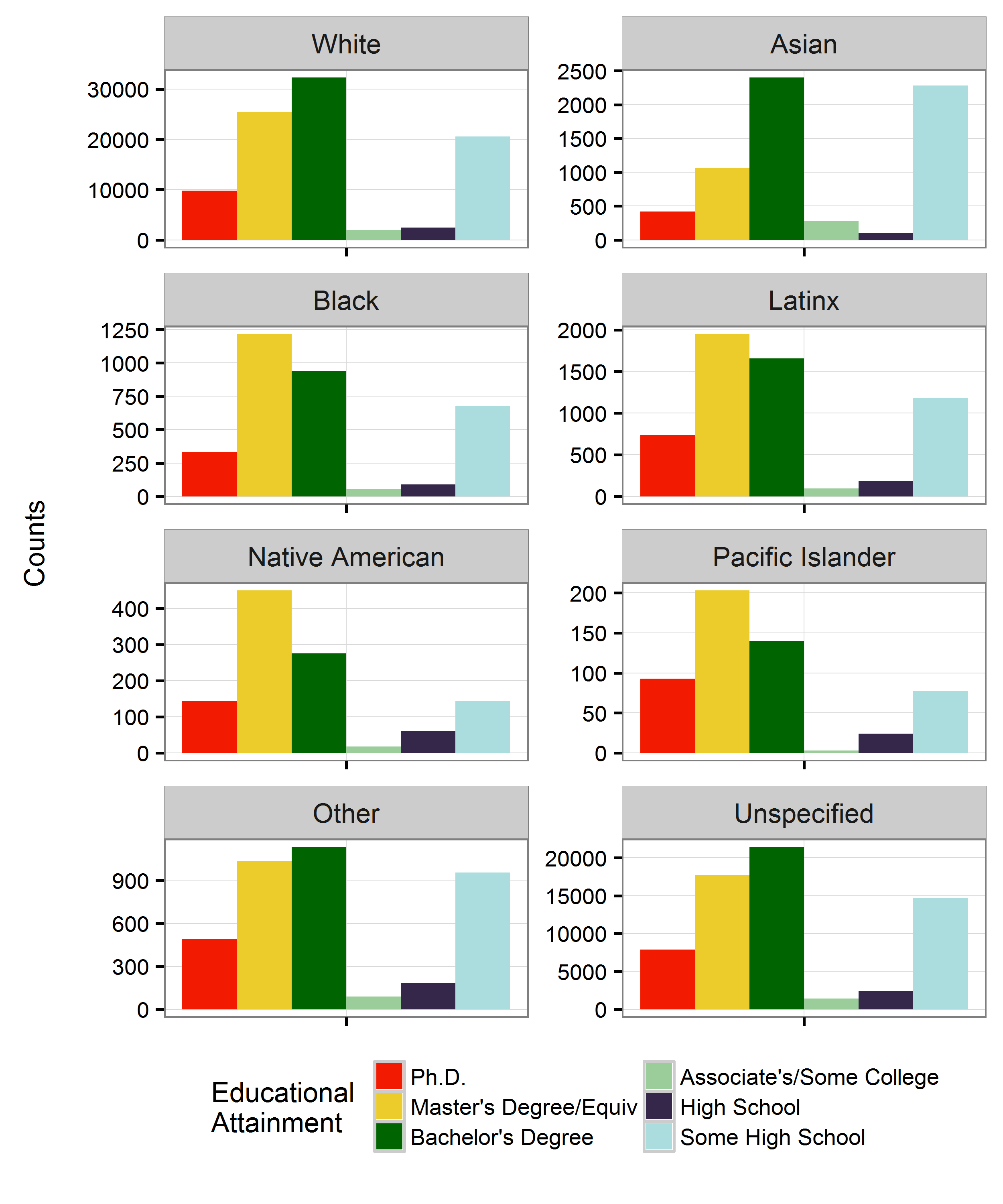

Supplement: S3 Fig — The number of participants across ethnic categories, reporting educational attainment between Some High School to Ph.D.’s at T1 (N = 196,388). (TIFF) [file pone.0182276.s003.tiff]

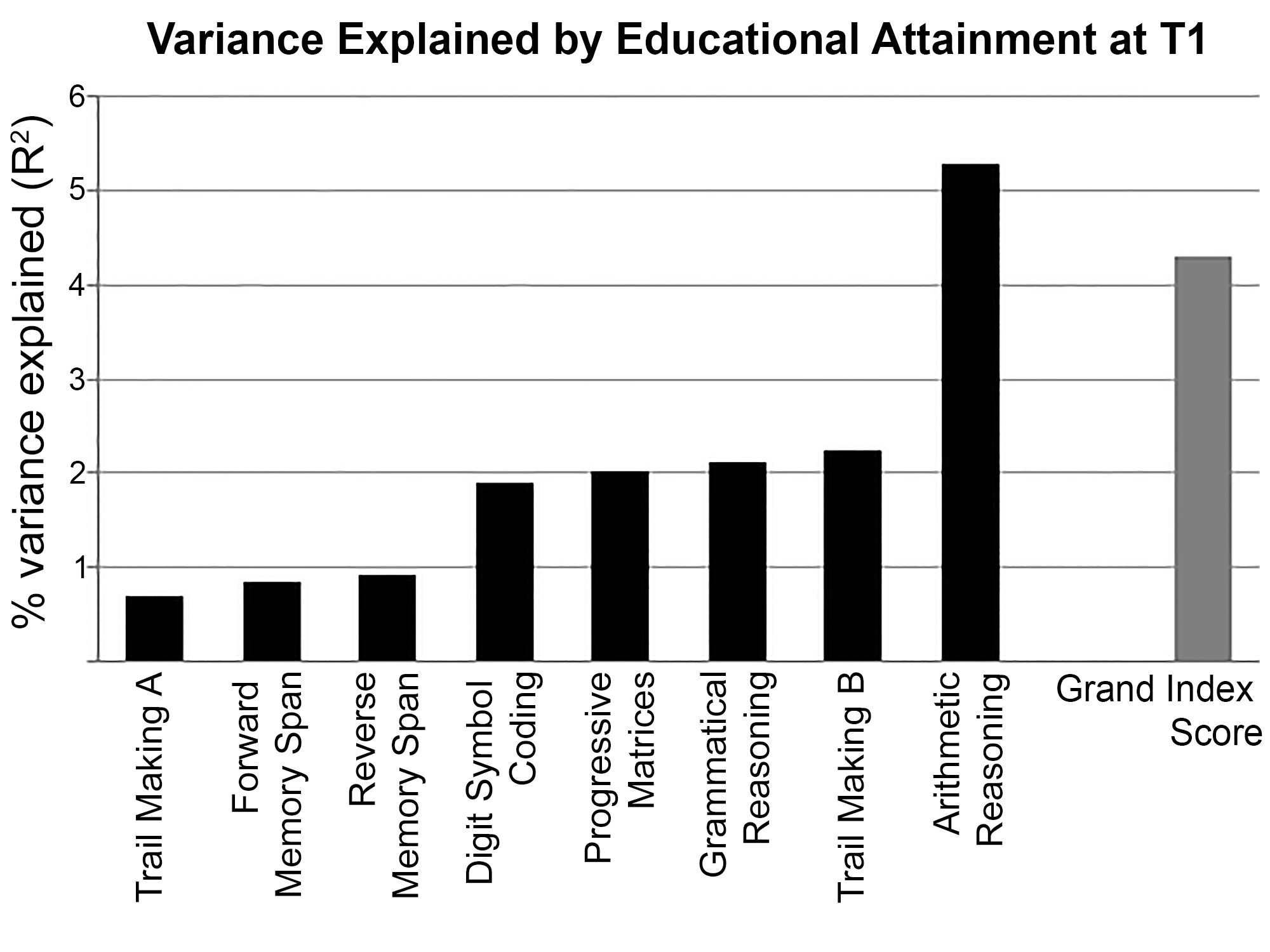

Supplement: S4 Fig — Displaying R2 values from regression models of educational attainment predicting performance on individual cognitive assessments. (TIFF) [file pone.0182276.s004.tiff]
