# Supplementary material for: Does higher education hone cognitive functioning and learning efficacy? Findings from a large and diverse sample
Source: PLoS One. 2017 Aug 23;12(8):e0182276. doi: 10.1371/journal.pone.0182276 (PMC5568102; doi:10.1371/journal.pone.0182276)
Supplement: S1 Table — (PDF) [file pone.0182276.s005.pdf]

S1 Table. Demographic and engagement covariates predicting T1 cognitive performance and learning, as measured by the Grand Index score (GI)

Covariate Type		GI T1 Estimate	Δ GI Estimate
	Intercept	100.69***	6.83***
**Engagement**	Lumosity game play time (h)	2.67***	1.37***
	Days between T1 and T2		-2.51***
**Native language**	English not native language	-5.77***	-0.52***
	Native language unspecified	-0.65**	-0.52**
**Gender**	Female	-3.79***	-0.80***
	Gender unspecified	-3.60***	-0.23
**Ethnicity**	Asian	5.63***	0.43**
	Black	-7.08***	-1.26***
	Latinx	-1.98***	-0.57***
	Native American	-2.16***	-0.36
	Pacific Islander	-0.21	0.58
	Other	-2.88***	-0.39*
	Unspecified	-1.18***	0.10
**Household Income**	0-25k	0.20	0.23
	25-50k	0.16	0.09
	50-75k	0.25	0.09
	100-125k	0.22	0.15
	125-150k	0.70**	0.15
	150-200k	0.97***	0.06
	200-250k	0.77**	-0.01
	Over 250k	0.83***	0.38*
	Non US income	0.74***	0.50**
	Unspecified	-1.01***	0.05
**T1 Performance**			-0.10***

Together, all these variables account for 5% of the variance in cognitive performance at both timepoints. It is plausible that the race effects are confounded by childhood SES, which we did not measure. The GI change score (ΔGI) is the difference between the GI score from T1 and T2. Each GI score was normalized to have a distribution with mean of 100 (15 SD) Reference categories: English speaker, Male, White, 75-100k household income. The logarithmic form of the engagement variables was used in the analyses. $p < 0.0001$‘***’, $p < 0.001$‘**’, $p < 0.01$‘*’
